# Supplementary figures and images for: The prolactin receptor scaffolds Janus kinase 2 via co-structure formation with phosphoinositide-4,5-bisphosphate
Source: eLife. 2023 May 26;12:e84645. doi: 10.7554/eLife.84645 (PMC10260020; doi:10.7554/eLife.84645)

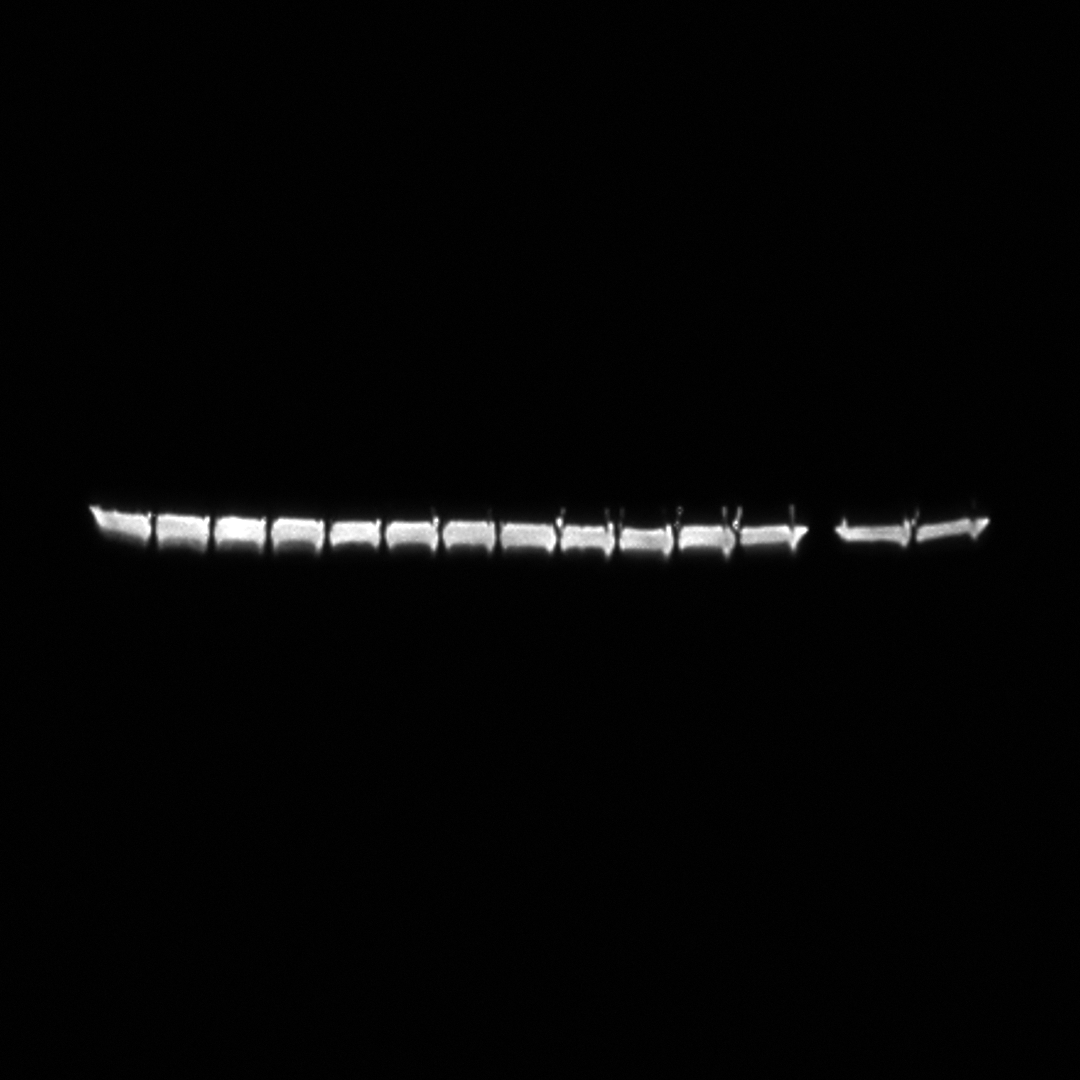

Supplement: Figure 5—source data 1. [file elife-84645-fig5-data1.zip › EXP9 1n 26.5.19 AP b-actin 0.4sec.Tif]

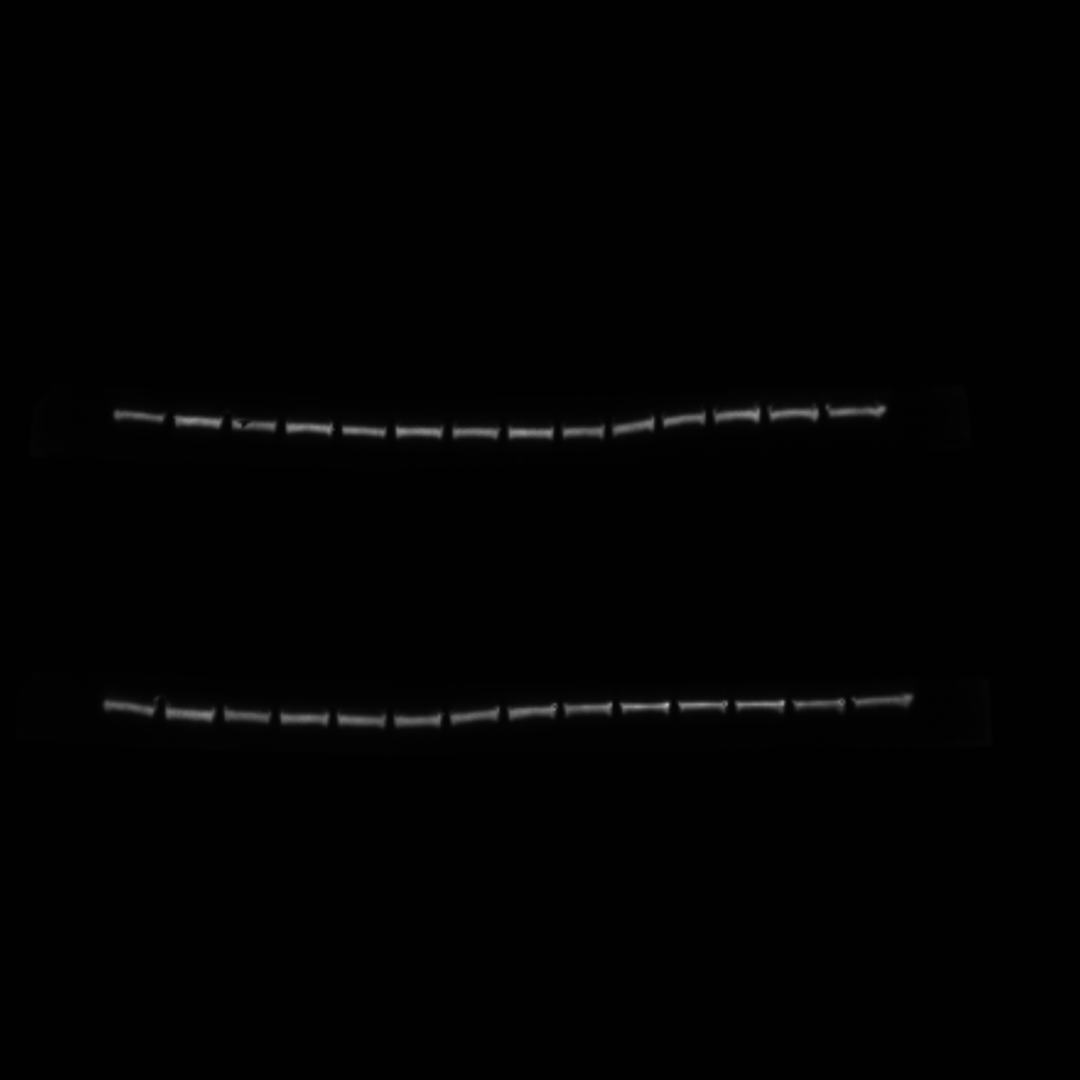

Supplement: Figure 5—source data 1. [file elife-84645-fig5-data1.zip › EXP9 1n 26.5.19 AP p150 4 sec.Tif]

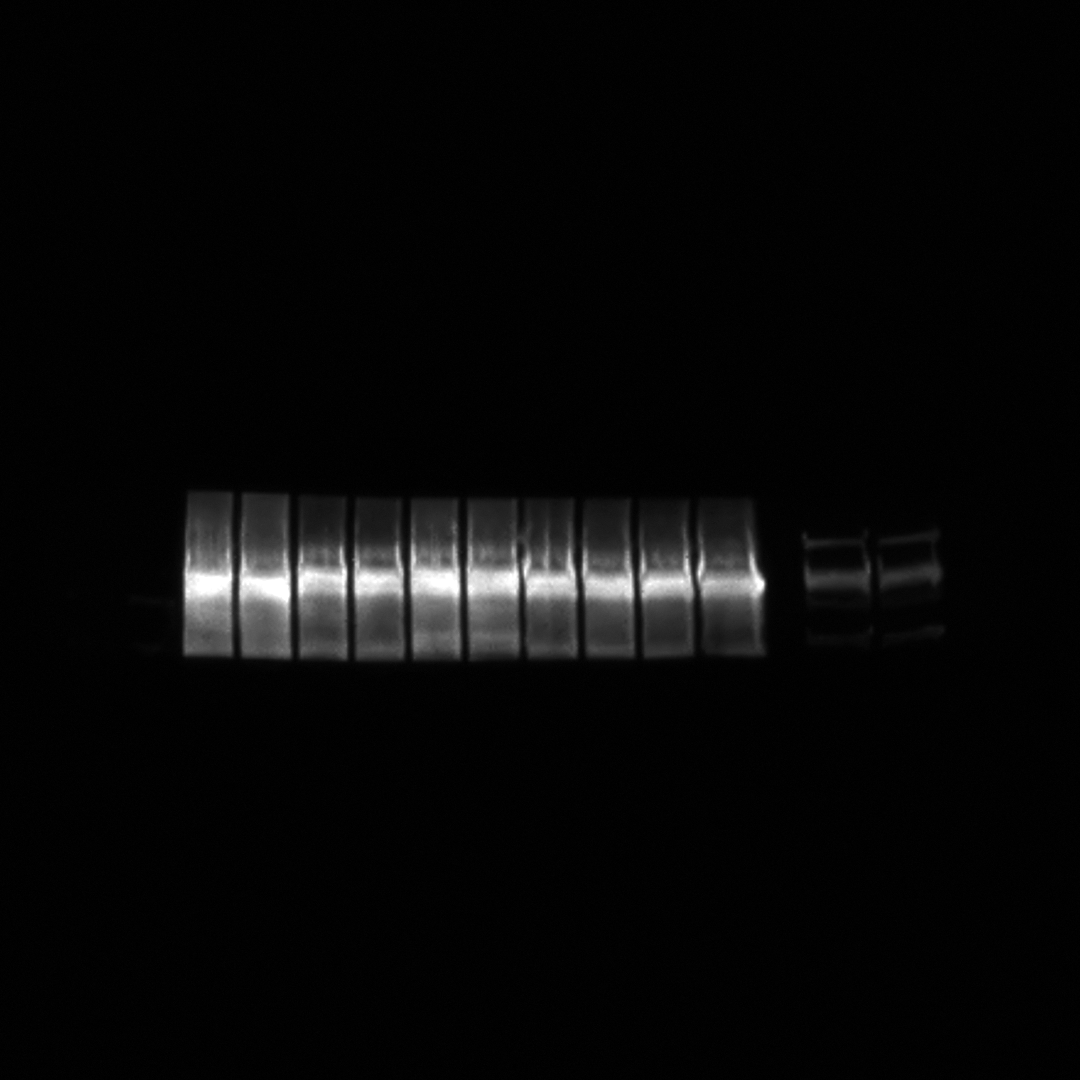

Supplement: Figure 5—source data 1. [file elife-84645-fig5-data1.zip › EXP9 1n 26.5.19 AP PRLR 2sec.Tif]

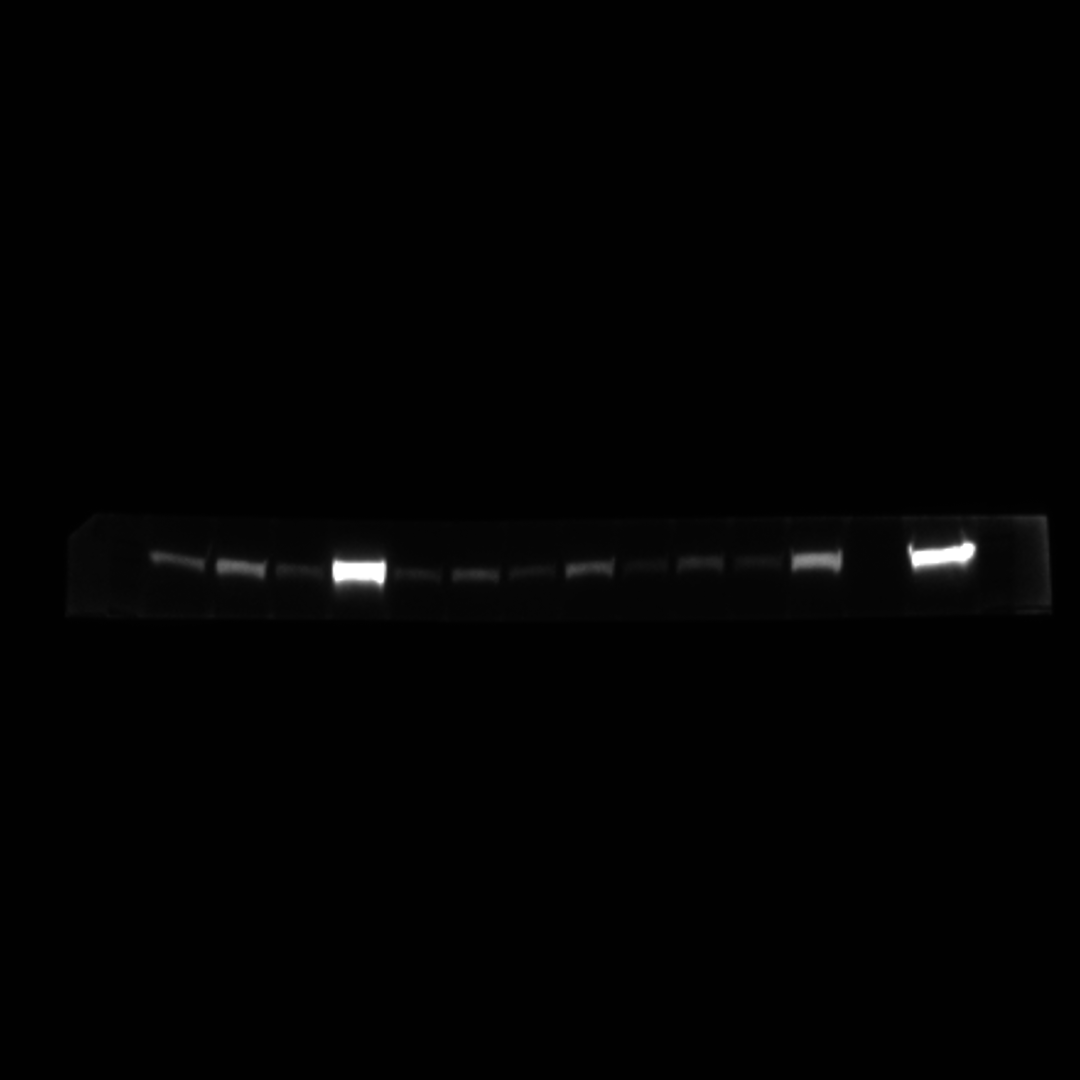

Supplement: Figure 5—source data 1. [file elife-84645-fig5-data1.zip › EXP9 1n 26.5.19 AP pSTAT5 30sec.Tif]

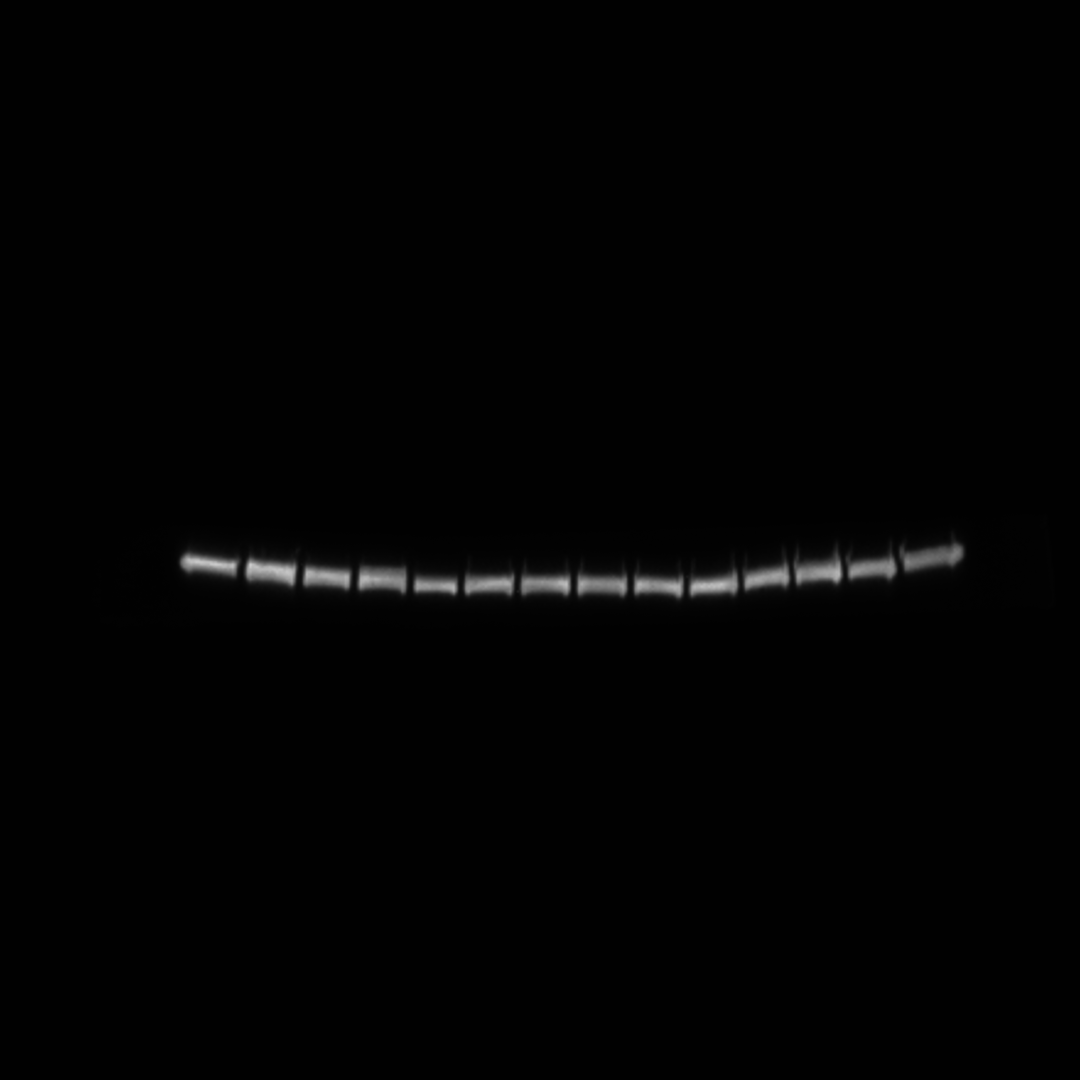

Supplement: Figure 5—source data 1. [file elife-84645-fig5-data1.zip › EXP9 1n 26.5.19 AP STAT5 4sec.Tif]

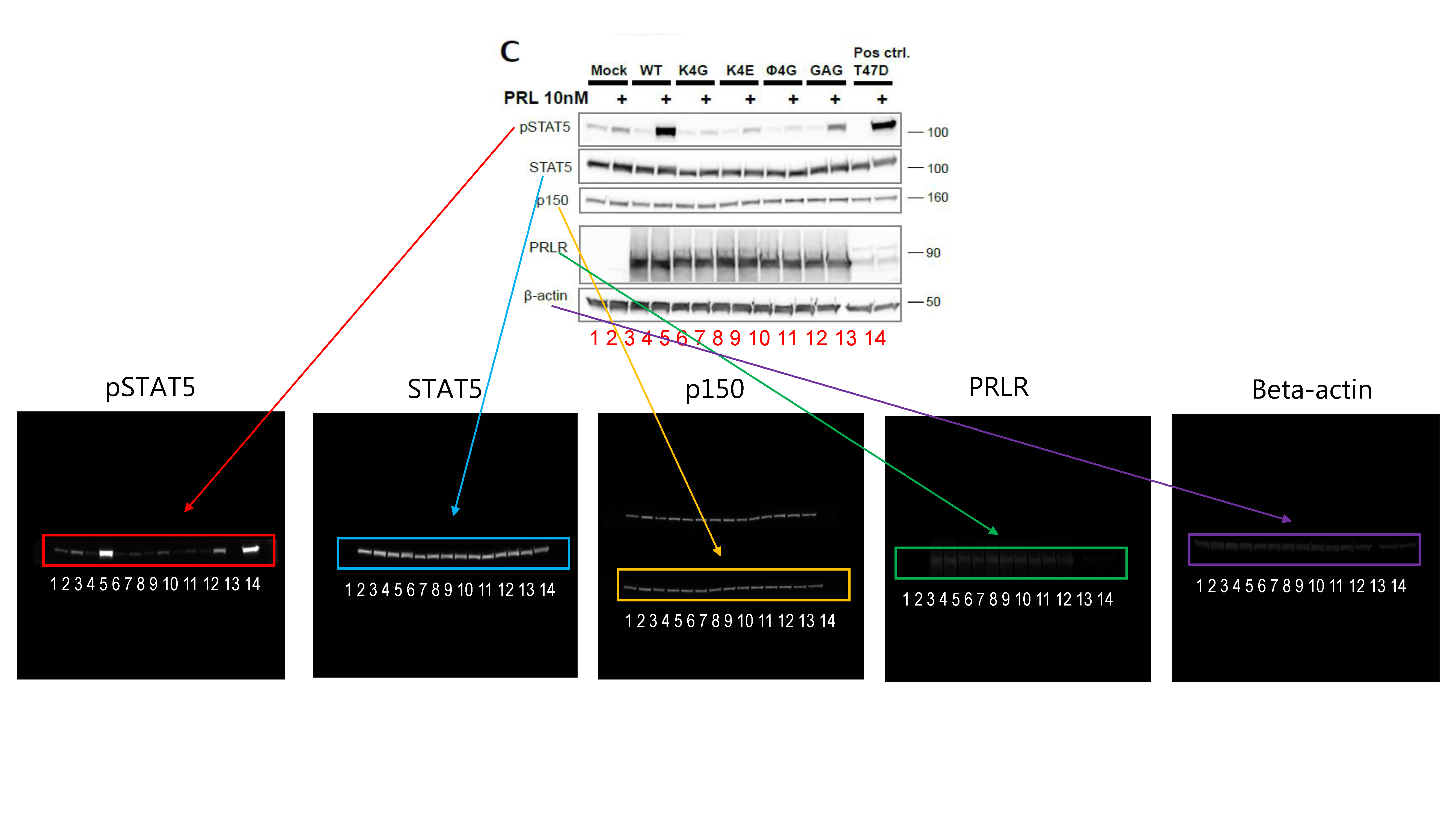

Supplement: Figure 5—source data 1. [file elife-84645-fig5-data1.zip › Fig 5C source data identification_2022-11-22.tif]

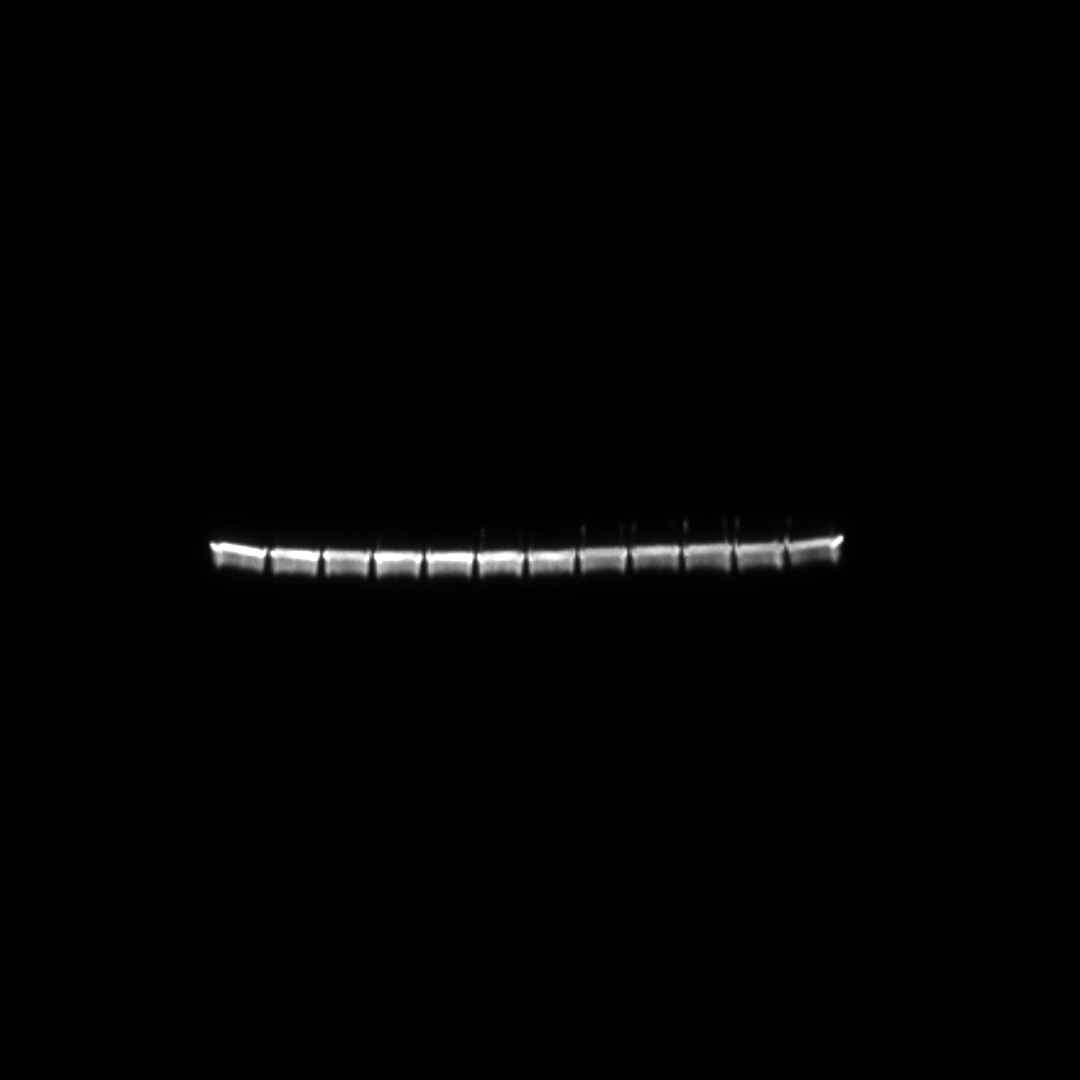

Supplement: Figure 5—source data 2. [file elife-84645-fig5-data2.zip › EXP12 1n 9.7.19 b-actin 0.4 sec.Tif]

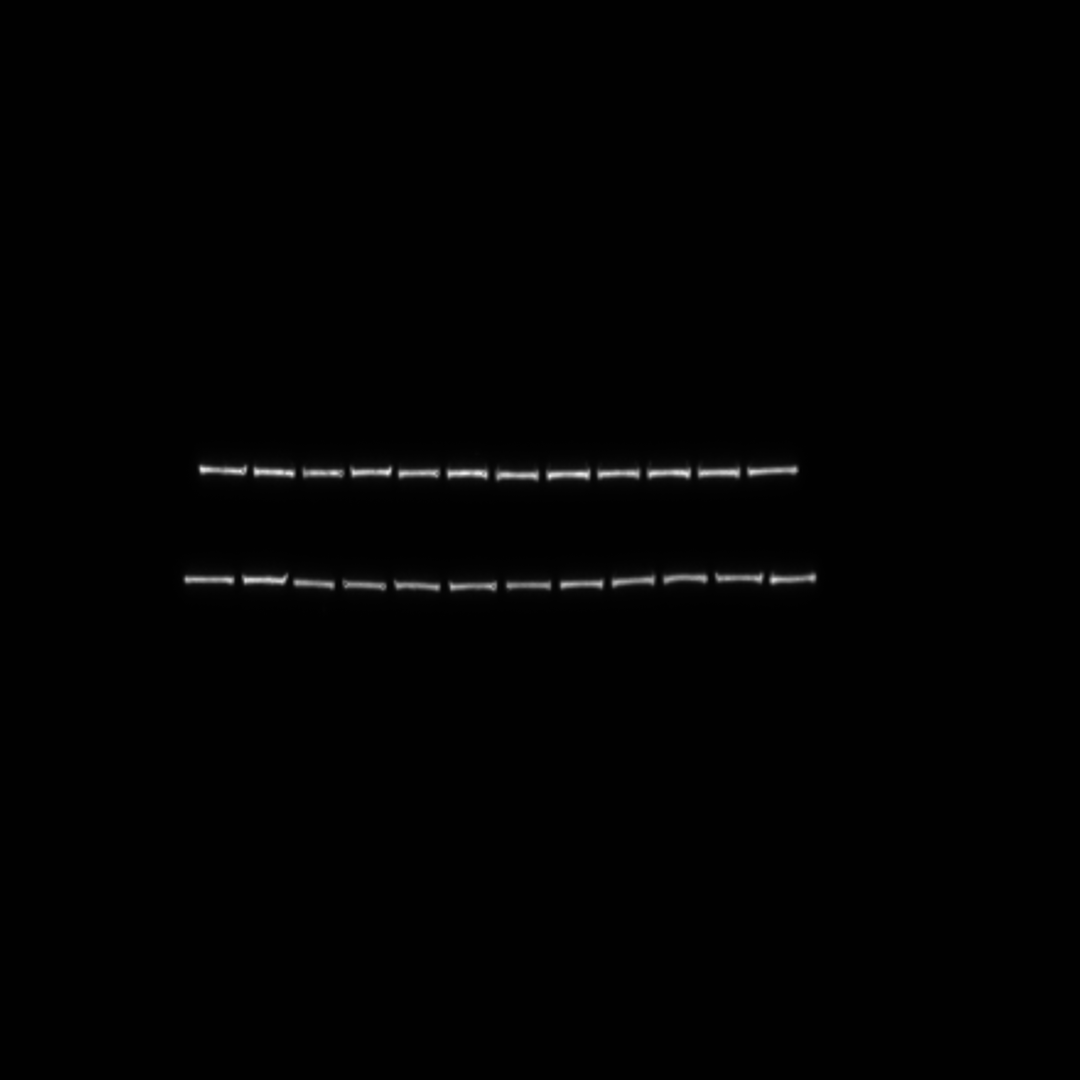

Supplement: Figure 5—source data 2. [file elife-84645-fig5-data2.zip › EXP12 1n 9.7.19 p150 5s .Tif]

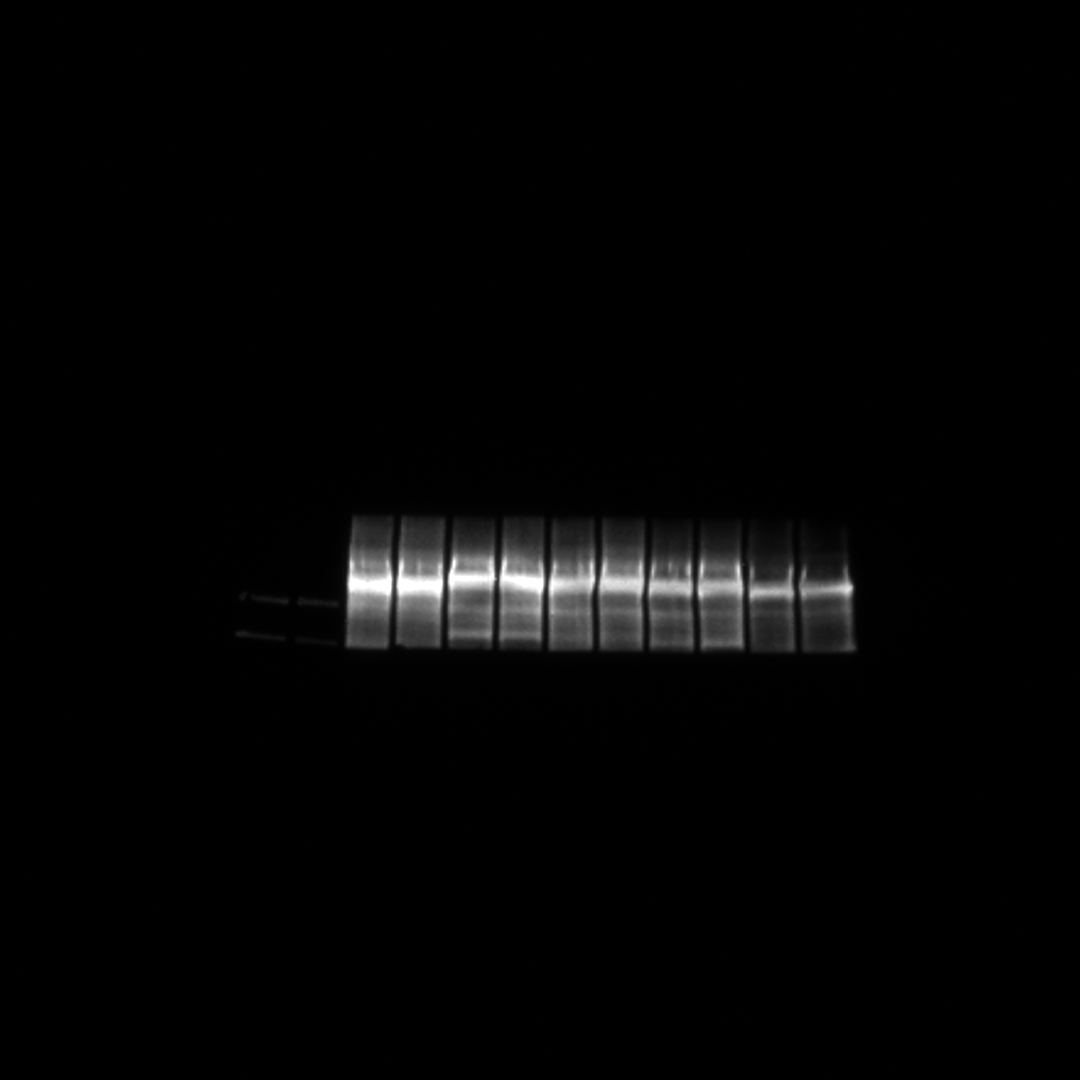

Supplement: Figure 5—source data 2. [file elife-84645-fig5-data2.zip › EXP12 1n 9.7.19 PRLR 1secTif.Tif]

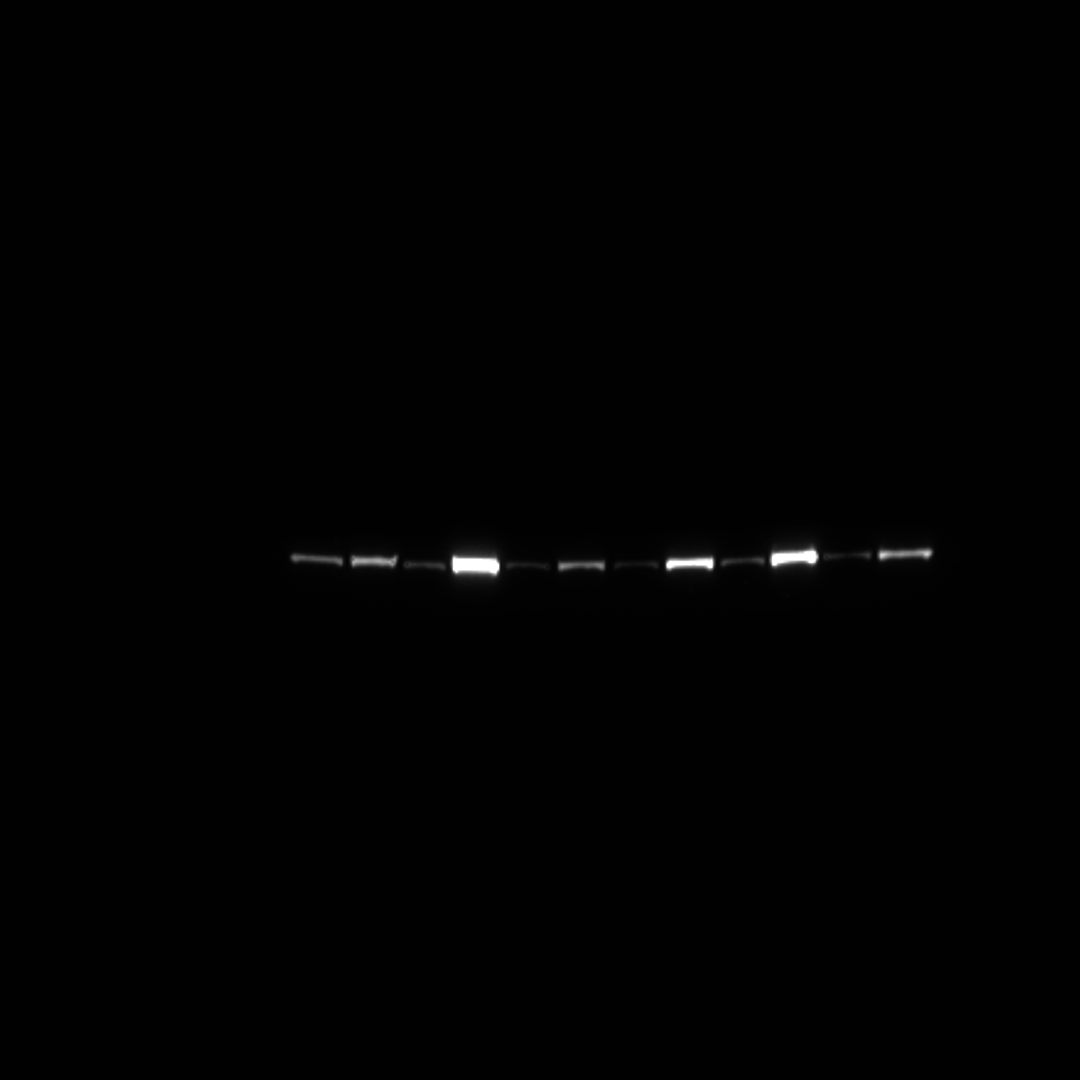

Supplement: Figure 5—source data 2. [file elife-84645-fig5-data2.zip › EXP12 1n 9.7.19 pSTAT5 20sec .Tif]

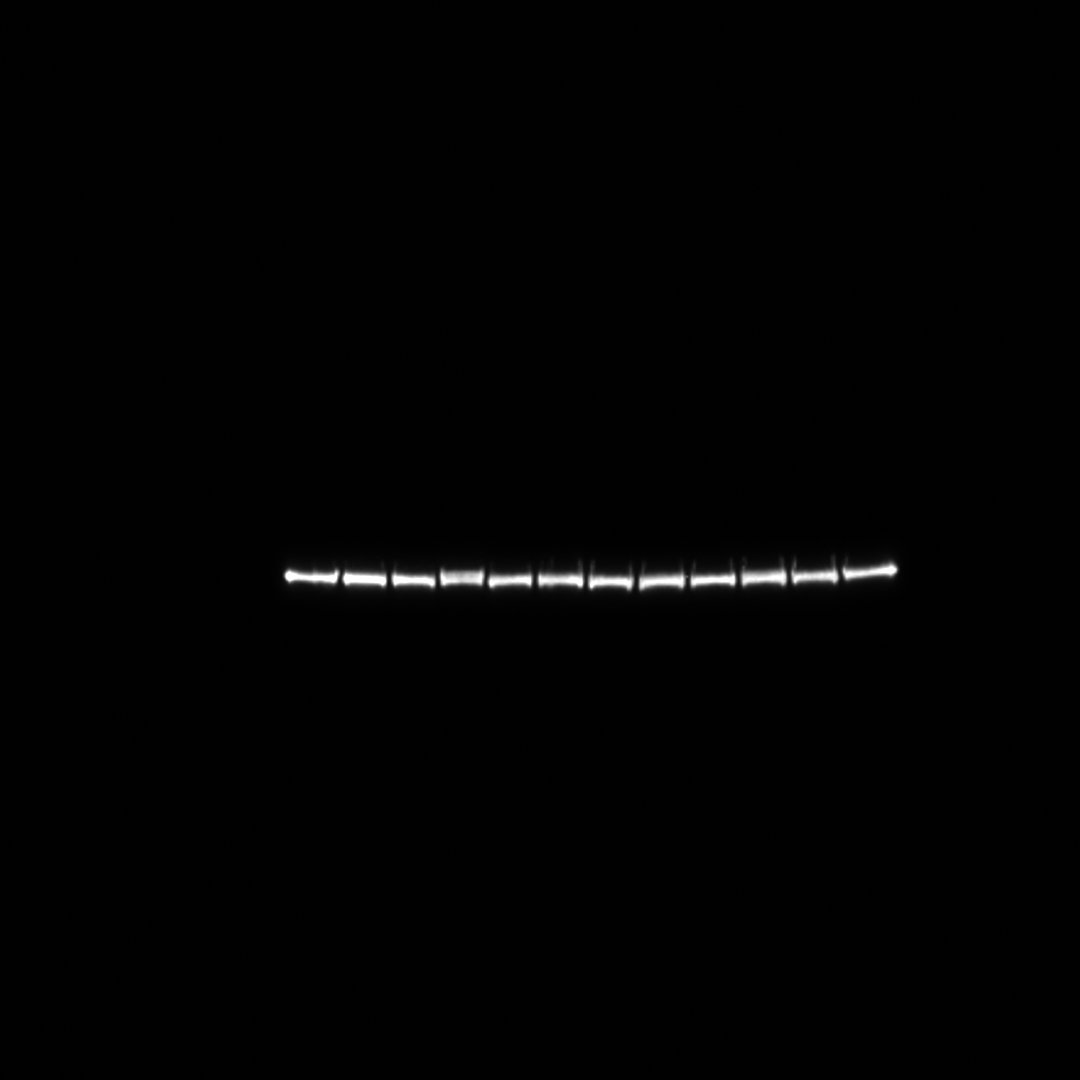

Supplement: Figure 5—source data 2. [file elife-84645-fig5-data2.zip › EXP12 1n 9.7.19 STAT5 3 sec .Tif]

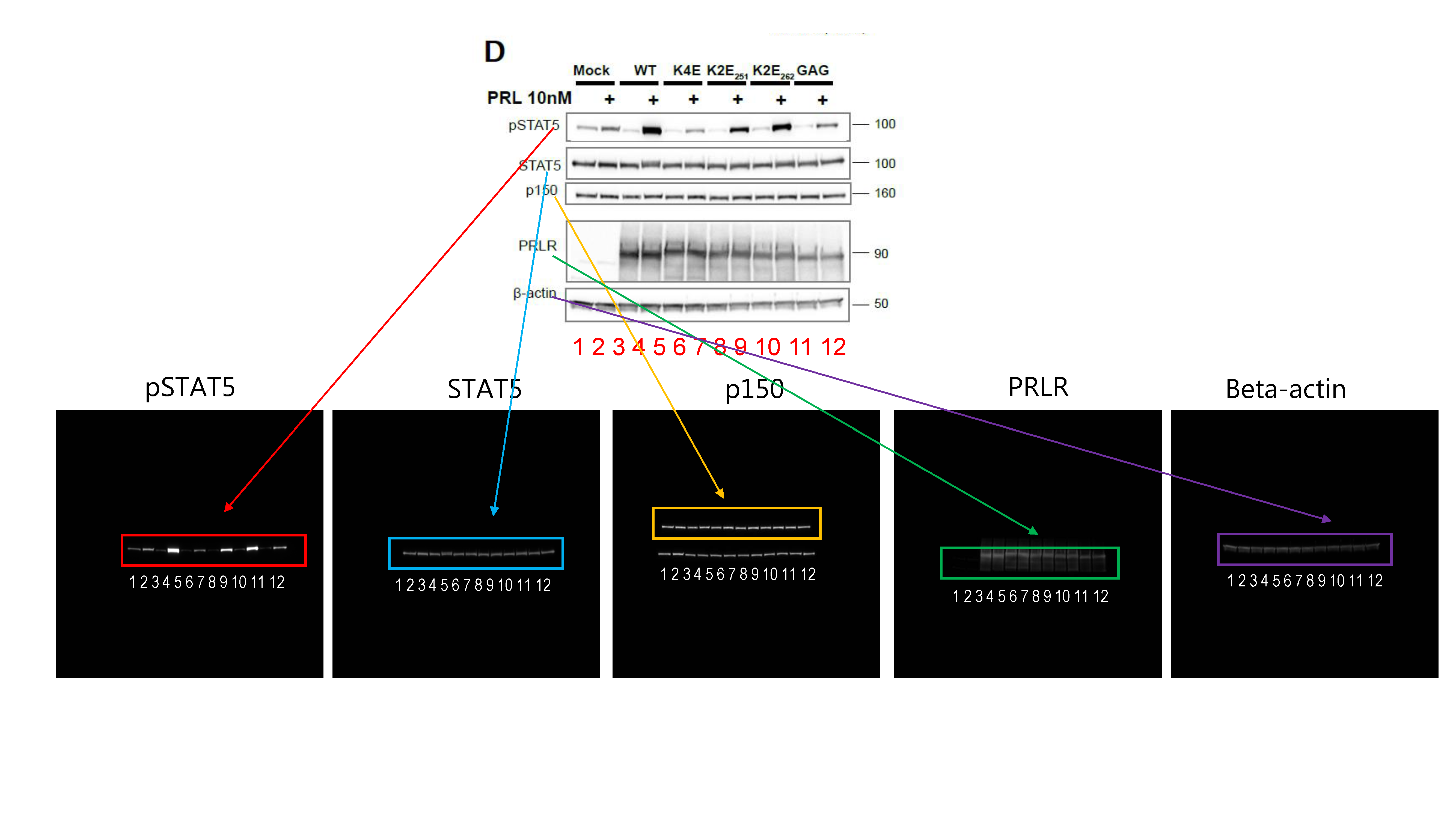

Supplement: Figure 5—source data 2. [file elife-84645-fig5-data2.zip › Fig 5D source data identification_2022-11-22.tif]

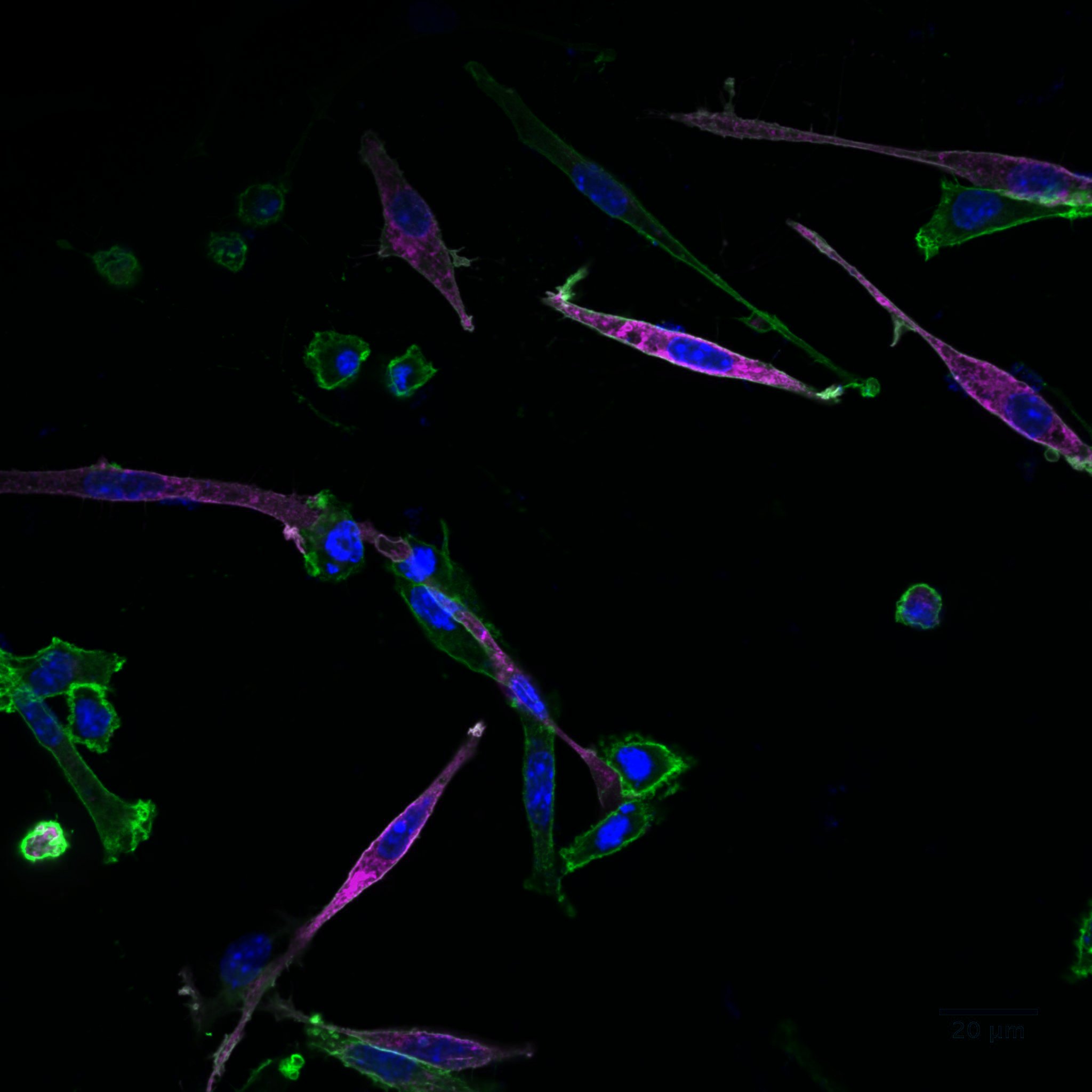

Supplement: Figure 5—source data 3. [file elife-84645-fig5-data3.zip › 3GAG merge.jpg]

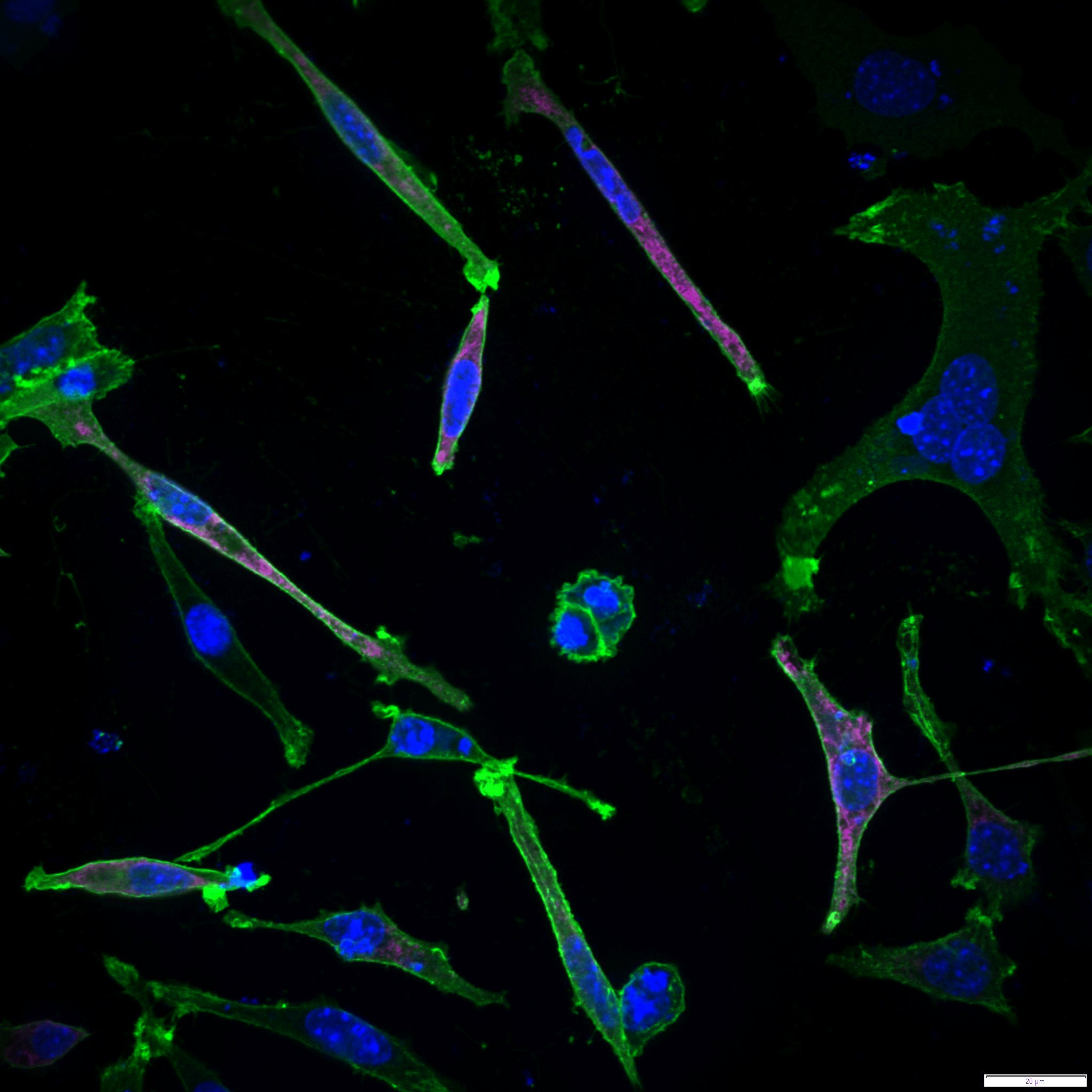

Supplement: Figure 5—source data 3. [file elife-84645-fig5-data3.zip › K4E merge.jpg]

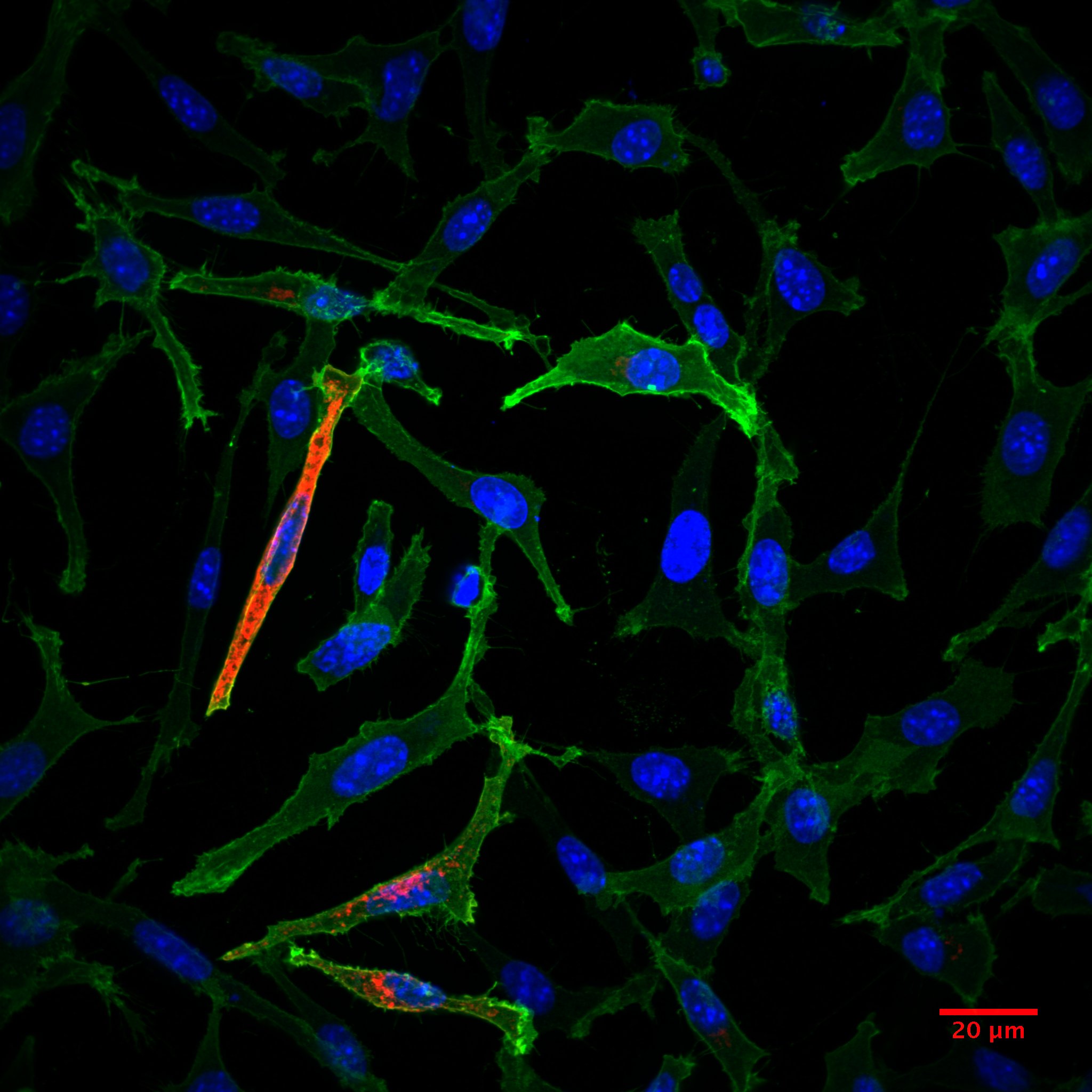

Supplement: Figure 5—source data 3. [file elife-84645-fig5-data3.zip › K4G merge.jpg]

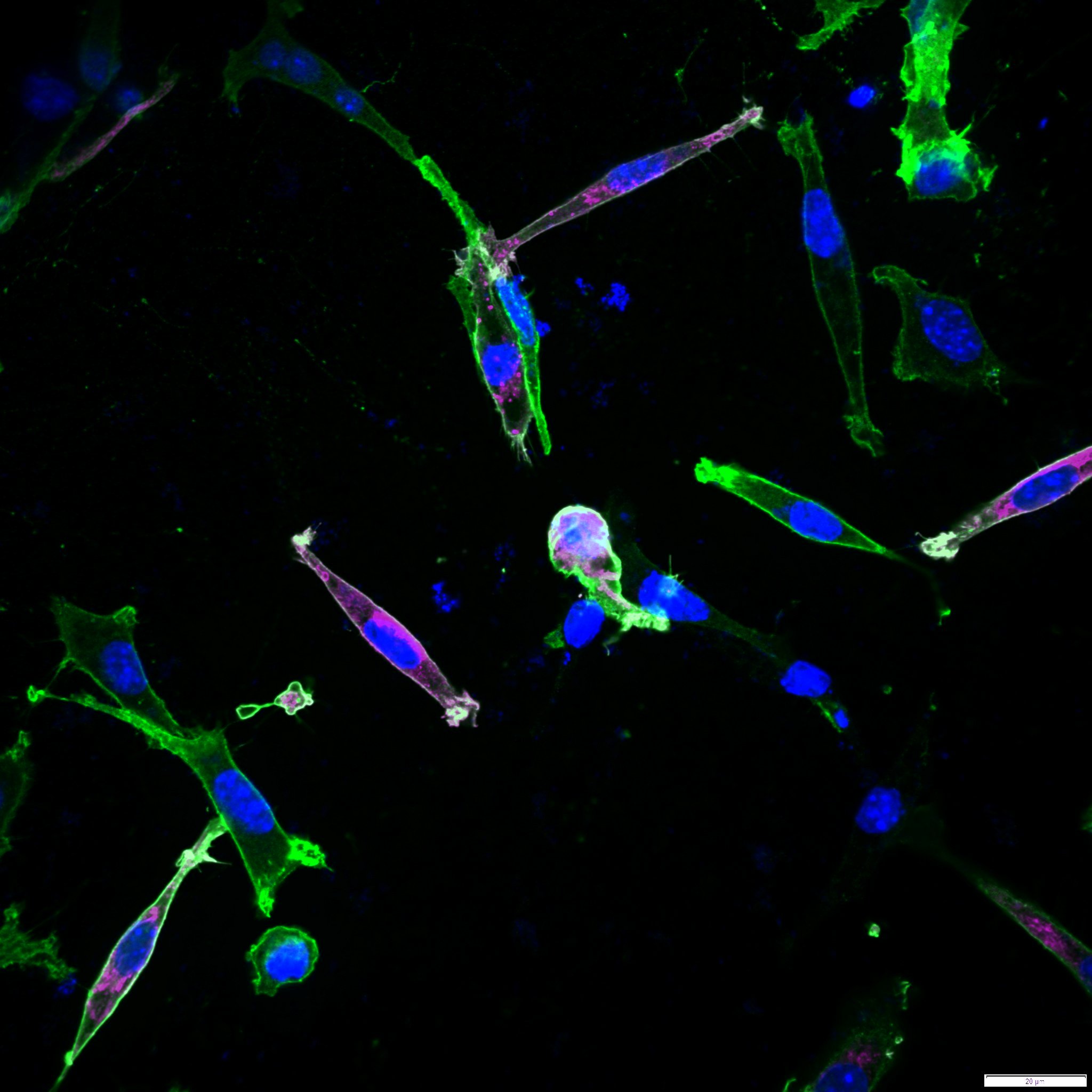

Supplement: Figure 5—source data 3. [file elife-84645-fig5-data3.zip › Phi4G merge.jpg]

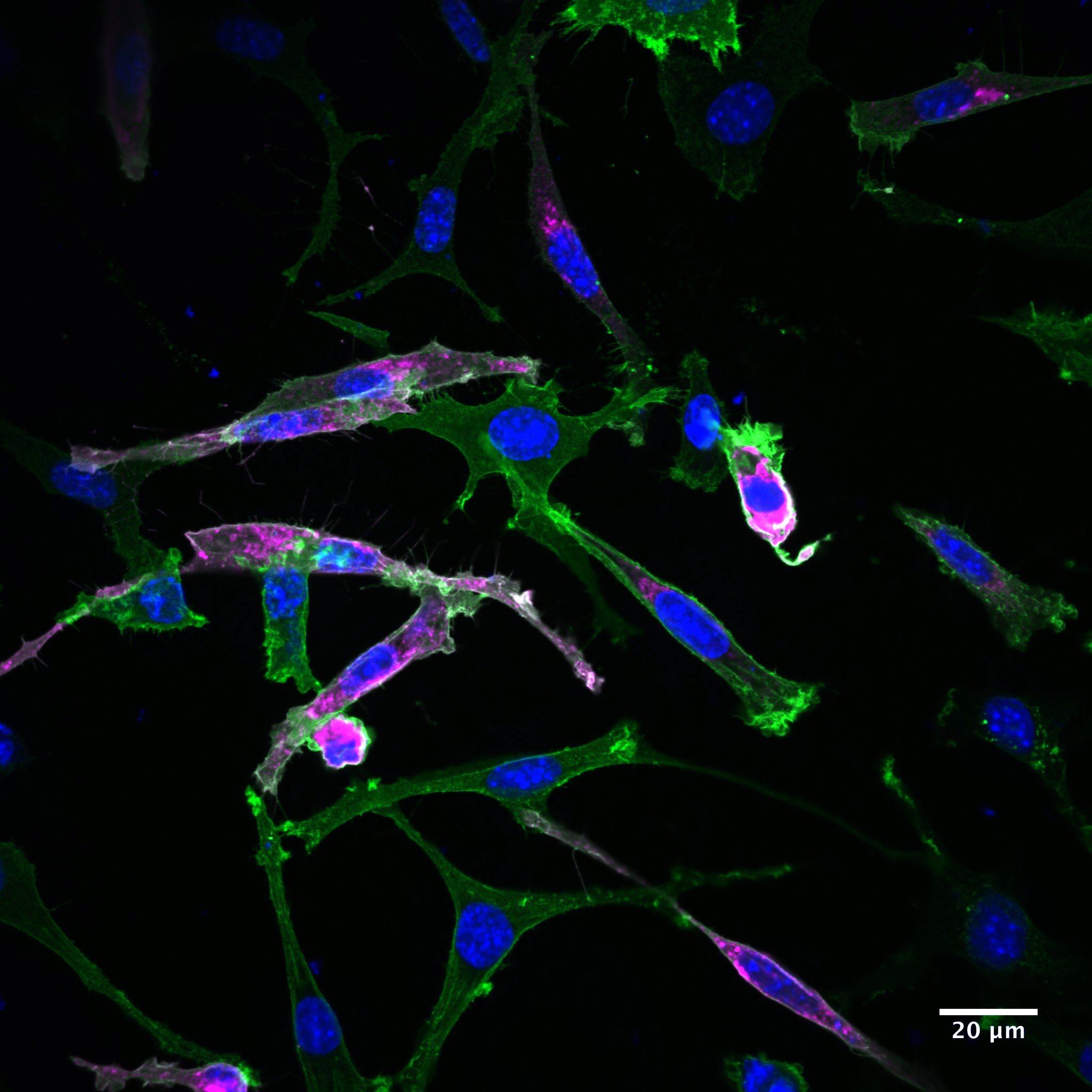

Supplement: Figure 5—source data 3. [file elife-84645-fig5-data3.zip › WT merge w. scalebar.jpg]
